# A Genetic Algorithm-Optimized ConvNeXtV2-YOLOv8 Framework for Dental Caries Detection in Panoramic Radiographs

**DOI:** 10.3390/diagnostics16152454

**Published:** 2026-08-03

**Authors:** Nebras Sobahi, Deniz Bora Küçük, Kazım Kılıç, Andaç İmak, Adalet Çelebi, Yazyd Alghamedi, Cafer Yazicioglu, Muammer Türkoğlu, Abdulkadir Şengür

**Affiliations:** 1 Department of Electrical and Computer Engineering, Faculty of Engineering, King Abdulaziz University, Jeddah 21589, Saudi Arabia; yalghamedi0003@stu.kau.edu.sa; 2 Department of Software Engineering, Computer and Information Sciences Faculty, Samsun University, 55000 Samsun, Turkey; deniz.kucuk@samsun.edu.tr (D.B.K.); muammer.turkoglu@samsun.edu.tr (M.T.); 3 Department of Artificial Intelligent Engineering, Computer and Information Sciences Faculty, Samsun University, 55000 Samsun, Turkey; kazim.kilic@samsun.edu.tr; 4 Department of Electrical and Electronic Engineering, Faculty of Engineering, Munzur University, 62000 Tunceli, Turkey; andacimak@munzur.edu.tr; 5 Oral and Maxillofacial Surgery Department, Faculty of Dentistry, Mersin University, 33000 Mersin, Turkey; adalet_celebi@hotmail.com; 6 Department of Construction, Vocational School of Technical Sciences, Batman University, 72000 Batman, Turkey; cafer.yazicioglu@batman.edu.tr; 7 Department of Electrical-Electronics Engineering, Faculty of Technology, Firat University, 23100 Elazig, Turkey; ksengur@firat.edu.tr

**Keywords:** dental caries detection, panoramic radiography, YOLOv8, ConvNeXtV2, genetic algorithm optimization, deep learning, object detection

## Abstract

**Background/Objectives:** Dental caries is one of the most common oral diseases worldwide, and early diagnosis is essential for effective treatment. However, detecting carious lesions in panoramic radiographs is challenging because of low image contrast, anatomical complexity, and overlapping structures. This study aimed to develop an improved object detection framework for dental caries localization in panoramic radiographs. **Methods:** A modified YOLOv8 architecture was developed by integrating ConvNeXtV2 blocks into the Cross-Stage Partial (C2f) modules to enhance feature extraction and gradient propagation. To improve detection performance and reduce overfitting, key training hyperparameters were optimized using a genetic algorithm. The proposed model was evaluated on a dataset of 474 panoramic dental radiographs annotated by experts using bounding boxes. **Results:** The optimized model achieved a box precision of 78.4%, a recall of 53.6%, and a mean average precision at 50% intersection-over-union threshold (AP50) of 62.6%. Compared with the baseline YOLOv8 model, the proposed approach improved precision and AP50. Visual and quantitative analyses demonstrated that the ConvNeXtV2-enhanced architecture enabled more accurate localization of carious regions. **Conclusions:** The results indicate that combining ConvNeXtV2-based architectural enhancement with genetic algorithm-based hyperparameter optimization is an effective strategy for dental caries localization in panoramic radiographs. Although recall remains a limitation, the proposed framework shows potential as a computer-aided diagnostic tool for supporting clinical caries assessment.

## 1. Introduction

Dental caries is a multifactorial disease characterized by damage to the hard tissues of the teeth (particularly enamel and dentin) resulting from a microbiologically driven process. The primary etiological factors include bacterial plaque adhering to the tooth surface, diet-derived fermentable carbohydrates (especially sugars), inadequate oral hygiene, and individual differences in host defense mechanisms [1]. These factors are evaluated within the framework of an interaction comprising the “four essential elements for caries”: the tooth surface (host), microorganisms, a suitable nutritional substrate (sugar, etc.), and time [2].

Tooth caries is one of the most common oral and dental health problems worldwide and can lead to serious consequences that reduce an individual’s quality of life [3]. Early diagnosis of caries is critical both for increasing the success rate of treatment and for preserving tooth structure [4,5,6,7,8]. While traditional methods for diagnosing caries include clinical examination, radiographic analysis, and visual inspection, the success of these methods often depends on the dentist’s experience, patient cooperation, and the quality of available equipment [5,8,9,10,11]. Additionally, the subjectivity involved in image interpretation and various diagnostic challenges lead to certain limitations regarding standardization and reliability in caries detection.

In recent years, rapid advancements in artificial intelligence (AI) and machine learning technologies have paved the way for innovative applications designed to support diagnostic processes in the field of dentistry [7,10,12]. In particular, deep learning approaches have proven highly successful in identifying meaningful patterns within large datasets, enabling the automatic detection of pathological conditions such as cavities, periodontal disease, or bone loss in radiographic images [7,10]. AI-assisted caries detection aims to enhance diagnostic accuracy and accelerate treatment planning for dentists by offering both time and cost efficiency [4,6,7]. Furthermore, this technological innovation, which seeks to improve patient safety and satisfaction, holds the potential to become an integral part of routine dental practice in the future [4,11]. In this context, various studies have been conducted in the literature on the segmentation and classification of caries [4,5,10]. Lasri and colleagues combined pre-trained CNN models (VGG-16, VGG-19, DenseNet-121, and Inception V3) with preprocessing techniques such as histogram equalization and Sobel edge detection on a dataset of 884 samples to detect dental caries using deep learning methods based on intraoral images; as a result of an optimization strategy incorporating Stochastic Gradient Descent (SGD) and Nesterov momentum, they achieved the highest accuracy of 98.3% using the VGG-16 model [9]. Thanh and colleagues conducted a comparative analysis of the Faster R-CNN, YOLOv3, RetinaNet, and SSD models using a dataset consisting of 1902 smartphone (iPhone 7) photos collected from 695 individuals; in this context, it was reported that while the YOLOv3 and Faster R-CNN models demonstrated sensitivities of 87.4% and 71.4%, respectively, in detecting cavitated caries, their sensitivity values dropped to 36.9% and 26% for visually non-cavitated lesions. Additionally, it was noted that the specificity values of these models for both lesion types ranged from at least 71% to 86% [12]. Casalegno and colleagues used a limited dataset consisting of only 185 near-infrared transillumination (TI) images for the early detection of dental caries. To this end, a Convolutional Neural Network (CNN)-based semantic segmentation model was developed by implementing measures to address the issues of data scarcity and class imbalance using various strategies. Achieving an average IoU of 72.7% in the five-class segmentation task, the model yielded IoU values of 49.5% and 49.0% for proximal and occlusal caries, respectively; in the simplified binary classification task, it achieved AUC values of 83.6% and 85.6% for occlusal and proximal lesions, respectively [4]. Lian and colleagues used an approach based on nnU-Net and DenseNet121 to detect carious lesions and classify them into different depth stages across 1160 panoramic dental X-rays. On this dataset, nnU-Net reported IoU and Dice values of 0.785 and 0.663, respectively, for caries segmentation, and demonstrated performance comparable to that of expert dentists in terms of accuracy and sensitivity, with values of 0.986 and 0.821. Additionally, DenseNet121 achieved accuracy rates of 0.957, 0.832, and 0.863, respectively, when classifying caries lesions according to their depths (D1: outer 1/3 of dentin; D2: middle 1/3 of dentin; D3: inner 1/3 of dentin), with sensitivity values for these stages recorded as 0.765, 0.652, and 0.918, respectively [5]. Khan and colleagues conducted a comparative analysis of various deep learning methods (CNN, LeNet, AlexNet, VGG16) using data obtained from the Khyber College of Dentistry and Hospital. In this study, the VGG16 model, which demonstrated the highest performance metrics, outperformed the other methods by achieving an accuracy of 98.99%, an F1 score of 0.96, a precision of 0.95, and a sensitivity of 0.97 [6]. Zhang and colleagues developed a Convolutional Neural Network (ConvNet) model based on the Single Shot MultiBox Detector (SSD) and trained it using a “hard negative mining” algorithm, using 3932 intraoral photographs obtained from 625 volunteers. The model achieved an AUC value of 85.65% in classifying the presence of caries, while sensitivity was reported at 81.90% per image and 64.60% per box (localization) at high sensitivity settings. It was emphasized that the “hard negative mining” approach, which has been shown to be effective in reducing false positives, statistically significantly improved both classification and localization performance (*p* < 0.001) [7]. Lee and colleagues developed a U-Net-based CNN model using 304 bitewing radiographs for the early detection of caries in posterior teeth; in tests conducted on 50 radiographs, the model achieved a precision of 63.29%, a recall of 65.02%, and an F1 score of 64.14% [8]. Imak and colleagues proposed a multi-input layered deep convolutional neural network ensemble (MI-DCNNE) model based on 340 periapical radiographs (including both carious and non-carious samples), using both raw periapical images and their enhanced versions as inputs. This approach, which applies the “score fusion” method in the model’s Softmax layer, achieved a 99.13% accuracy rate in classifying dental caries [10]. Oztekin and colleagues conducted a comparative analysis of pre-trained models such as EfficientNet-B0, DenseNet-121, and ResNet-50 using panoramic images obtained from 562 patients; these models generated heatmaps highlighting suspicious areas on the teeth in addition to classifying teeth as carried or non-carried. While the three models yielded similar results, ResNet-50 demonstrated slightly higher performance compared to the others, achieving 92% accuracy, 87.33% sensitivity, and an F1 score of 91.61 [11]. Haghanifar and colleagues aimed to detect dental caries by combining various pre-trained deep learning models and a capsule network architecture using transfer learning, based on 240 labeled images selected from a dataset of 470 panoramic X-rays. This approach achieved an accuracy rate of 86.05% on the test dataset, with sensitivity values of 69.44% for mild caries lesions and 90.52% for severe caries [13]. Furthermore, recent advances in hybrid architectures, such as transformer-based bilateral encoder–decoder networks, have demonstrated significant potential in addressing the challenges of tooth recognition and periodontal bone loss assessment in panoramic radiographs. These studies highlight that combining the global contextual modeling capabilities of transformers with the fine-grained local feature extraction of CNNs leads to more robust diagnostic outcomes in complex dental imaging scenarios [14].

These studies have achieved notable results, but direct comparison is difficult due to differences in imaging modality, annotation strategy, output type, and evaluation metrics. Classification-based methods can determine the presence of caries, but they generally do not provide direct lesion coordinates. Segmentation approaches offer pixel-level localization but require detailed, time-consuming annotations. Object detection methods strike a practical balance by localizing suspicious regions with bounding boxes. However, detecting caries in panoramic radiographs remains challenging due to low lesion visibility, overlapping anatomical structures, and variations in radiographic contrast.

To address these limitations, this study proposes an original ConvNeXtV2-enhanced, Genetic Algorithm-optimized YOLOv8 framework. Our approach transcends simple model stacking by embedding advanced ConvNeXtV2 blocks directly into selected C2f modules of the YOLOv8 architecture, thereby fundamentally strengthening its fine-grained feature extraction and representation capabilities. Furthermore, we implement a custom Genetic Algorithm-based hyperparameter optimization strategy to stabilize this complex hybrid architecture, ensuring a more reliable training configuration than that obtained through standard empirical settings. This design aims to improve YOLOv8’s localization capability while maintaining the practical advantages of bounding box-based object detection. The main contributions of this study are as follows:

- First, a ConvNeXtV2-enhanced YOLOv8 architecture was proposed to automate the detection of dental caries in panoramic radiographs.
- ConvNeXtV2 blocks were integrated into selected C2f modules to enhance feature representation in low-contrast and anatomically complex regions.
- An optimization strategy based on genetic algorithms is applied to determine effective training hyperparameters, including the learning rate, momentum, weight decay, and warmup-related parameters.
- A dataset consisting of 474 panoramic radiographs, collected from clinical settings, is annotated by dental experts using bounding boxes and converted into YOLO format.
- We compared the proposed model with the baseline YOLOv8 model and the non-optimized ConvNeXtV2-enhanced model using the Box Precision, Recall, and AP50 metrics.
- We conduct quantitative, qualitative, confidence threshold, and limitation analyses to evaluate the model’s detection behavior under challenging panoramic radiographic conditions.

## 2. Materials and Methods

In this study, we present an enhanced YOLOv8 object detection model with ConvNeXtV2 blocks for automatically detecting and localizing dental caries in panoramic radiographs. The low contrast, limited resolution, and superposition of anatomical structures in panoramic radiographs make it difficult to detect caries, especially small, early-stage lesions. The proposed approach combines YOLOv8’s fast, effective object detection capabilities with ConvNeXtV2 blocks’ advanced feature extraction capabilities. The methodological process consists of four main stages. These are:

- In the first stage, experts annotated 474 panoramic radiographic images obtained from a clinical setting using the bounding box technique, and the annotations were converted to YOLO format. This process ensured the model would focus on caries regions during training and standardized the data format.
- In the second phase, YOLOv8 was used as the base object detection architecture. Thanks to its single-stage structure, YOLOv8 can directly identify carried regions in panoramic images using bounding boxes.
- In the third stage, ConvNeXtV2 blocks were integrated into specific C2f modules of the YOLOv8 architecture to improve the model’s ability to extract features. This integration aimed to represent low-contrast and morphologically ambiguous carry patterns with more distinctive features.
- In the fourth stage, genetic algorithm (GA)-based hyperparameter optimization was applied to improve the training performance of the proposed architecture. The initial learning rate, momentum, weight decay, and warmup parameters were evaluated across a wide search space to determine the most suitable hyperparameter combination for the model. The goal was to stabilize the training process and improve the precision–recall balance.

**Figure 1:**
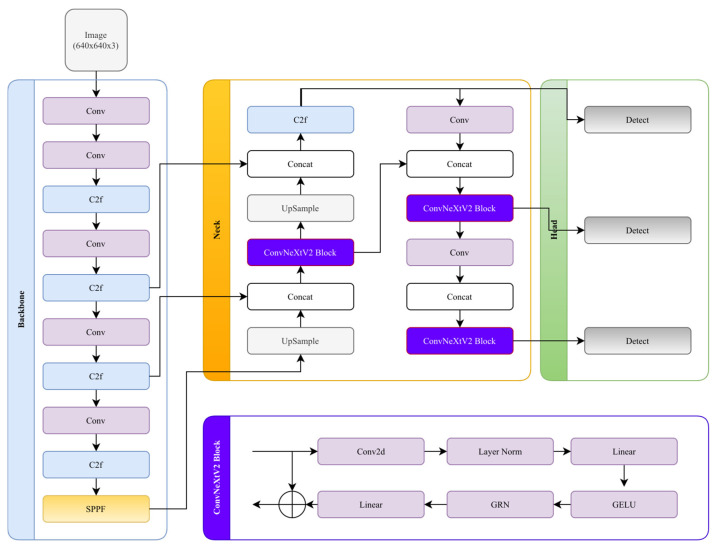
General workflow of the proposed ConvNeXtV2-enhanced and GA-optimized YOLOv8 architecture.

Finally, we evaluated the performance of the proposed model in comparison with the standard YOLOv8 model and the unoptimized version of the proposed model. The evaluation process used the box precision, recall, and AP50 metrics. These metrics enable an analysis of the model’s tendency to generate false positives, its ability to detect defective regions, and its overall localization performance. Figure 1 presents the general workflow of the proposed architecture.

As shown in Figure 1, the architectural flow consists of data preparation, a YOLOv8-based object detection framework, architectural improvements using ConvNeXtV2 blocks, and GA-based hyperparameter optimization. This integrated framework provides an approach to support decision-making aimed at detecting various areas in panoramic radiographs in a more balanced and reliable manner. The algorithms and architectural components used are described in detail in the following subsections.

### 2.1. YOLOv8 Architecture

In CVPR 2016, Joseph Redmon et al. propose a real-time end-to-end approach for the first time with the YOLO (You Only Look Once) model [15]. YOLO, compared to other models in object detection, has the ability to detect all objects with a one-time analysis of the image. When other object detection methods are examined, models generally perform the detection process by working on each image many times, thanks to sliding window algorithms together with the classifier. RCNN systems were developed, and the transition to two-stage methods was provided with the use of region proposal networks in subsequent stages. The Fast R-CNN [16] model performs the classification for the proposals of object regions on the image in two stages, with the bounding box and classification process in the second stage. Unlike these models, YOLO uses a simpler output with a single network pass to determine both the class label of the object and the bounding box location [17].

The YOLOv8 model, developed by the Ultralytics team for the object detection task, was proposed in 2023 [18]. With the success of the team’s YOLOv5 model, more advanced architectural features, which stand out in YOLOv8, have been developed by integrating feature extraction and fusion processes. Among its basic features, YOLOv8 uses a more advanced version of the CSPDarknet backbone [19]. Cross Stage Partial (CSP) networks include dense blocks, a transition layer, and fusion features, a structure that improves gradient flow and reduces computational complexity. YOLOv8 also uses the SiLU (Swish) activation function, which has advantages such as faster convergence and improving gradient flow, instead of the traditional ReLU and Leaky ReLU that are generally used as activation functions. In addition, the network architecture includes the C2f (Cross Stage Partial with two fusion) building block [20]. Thanks to the C2 block, it allows the separation of complex patterns and the extraction of fine-grained features. The x feature map coming from the previous layer can be expressed mathematically as given in Equation (1) in the C2f module as follows [21].

(1) $${CV1}_{output}=Conv(c1,\;2\times c,1,\;1)(x),$$

Here, *Conv*(*c*1, *c*2, k, s), Conv is a convolution operation, c1 is the number of input channels, c2 is the number of output channels, k = 1 (1 × 1) is the kernel size and s = 1 is the stride size. The next step is given in Equation (2). In *Conv*(*c*1, *c*2, k, s), *Conv* is a convolution operation, *c*1 is the number of input channels, *c*2 is the number of output channels, k = 1 (1 × 1) is the kernel size and s = 1 is the stride size. The next step in Equation (2):

(2) $$y=Chunk({CV1}_{output},\;2,\;1),$$

Here, in *Chunk*(*x*, *n*, *d*), Chunk allows the tensor to be divided into pieces in the specified dimension. It expresses the division into n pieces along the D dimension. At the end of this process, the tensor is divided into two tensors, *y*1 and *y*2. In Equation (3), the next step for each *m* in ModuleList:

(3) $$y=Extend(y,\;m\left(y\left[1\right]\right)),$$

Here, m represents any layer operation, and Extend allows adding the output of *m*(.) operations to tensor y. Finally, in Equation (4):

(4) $${CV2}_{output}=Conv((2+n)\times c,c2,\;1)(Concate(y,\;1)),$$

Here, *Concate* allows the merging of parts of the tensor. Also, the computational cost is reduced by the pointwise convolution and decomposition operations in the Pointwise C2f module. YOLOv8 is a remarkable module that enhances the feature representation by integrating the innovative C2f module and the spatial pyramid pooling fractional (SPPF) approach [22].

YOLOv8 aims to improve object detection performance by increasing the accuracy of finding and sizing bounding boxes using Complete Intersection over Union (*CıoU*) and Distribution Focal Loss (DFL). The *CIoU* loss function is mathematically expressed as given in Equation (5) as follows:

(5) $$L_{CIoU}=1-IoU+\frac{\rho^{2}\left(b,\;\;b^{gt}\right)}{c^{2}}+\alpha v,\;\;\alpha =\frac{v}{1-IoU+v},\;v=\frac{4}{\pi^{2}}{\left(arctan\frac{w^{gt}}{h^{gt}}-arctan\frac{w}{h}\right)}^{2},$$

Here, *IoU* represents intersection over union. $\rho^{2}\left(b,\;b^{gt}\right)$ represents the distance between two points, estimated and real center points, respectively. c represents the minimum limiting diagonal distance of the box, *v* represents the width and height ratio consistency, while w and h inside represent the predicted width and height, respectively. Finally, α represents the weight coefficient obtained at a certain rate [23].

### 2.2. ConvNeXtV2 Block

ConvNeXtV2 Blocks constitute one of the fundamental steps in the main line for obtaining features. The ConvNeXt series is a model designed to address the efficiency of convolution operations by including advanced structures inspired by transformer models. Firstly, it plays an important role in capturing large spatial features with extended kernel structures in the ConvNeXtV2 block. Secondly, a normalization layer is used to stabilize the training processes [24]. Additionally, instead of the traditionally used ReLU layer to improve gradient flow, it includes the GELU activation function to produce values close to zero for negative data. As shown in Figure 1, the tensor obtained by using the Conv2d convolution layer used for the input $X\in R^{H\times W\times C}$ can be expressed as in Equation (6):

(6) $$Y_{j}^{l}=f\left(\sum_{i\in M_{j}}X_{i}^{l}\cdot k_{ij}^{l}+b_{j}^{l}\right),$$

As can be seen here, the result is obtained by adding the bias $b_{j}^{l}$ to the feature map after the pointwise multiplication of the input tensor $X_{i}^{l}$ with the kernel $k_{ij}^{l}$ in a specific dimension. Finally, the output tensor $Y_{j}^{l}\in R^{Hout\times Wout\times Cout}$ is produced by applying the activation function *f*(.) [25]. The obtained feature map is passed through the Layer normalization layer used to stabilize the training process in the second step and the linear and gelu activation functions to strengthen the feature expression. In the next stage, it is subjected to the global response normalization (GRN) process, which is one of the new basic features within the ConvNeXtV2 blocks. Thanks to the introduced GRN layer, it captures the dependencies between channels well in high-dimensional feature maps. In Equation (7), the layer normalization, activation and GRN processes are expressed as follows [24]:

(7) $$\hat{Y}=\frac{Y-\mu }{\sigma },\;Y_{function}=f(\hat{Y}),\;Y_{GRN}=\gamma .\frac{Y_{function}}{{\left\|Y_{function}\right\|}_{2}}+\beta ,$$

Here, Y is the feature map obtained from convolution layer. μ and σ represent the mean and standart deviation of the input tensor Y, and $\hat{Y}$ represents the normalized output. *f*(.) represents the activation function. γ is the scalimg parameter, β is the shift parameter, ${\left\|,\right\|}_{2}$ is the L2 norm, and $Y_{GRN}$ represents the normalized feature map. As can be seen in Figure 1, the residual block layer is used last. The residual block is expressed as in Equation (8).

(8) $$X_{out}=Y_{GRN}+X,$$

Here, *Xout* represents the final output. With these skip connections, the model also provides a solution to the vanishing gradient problem. ConvNeXtV2 blocks are integrated into the proposed model with many advantages.

### 2.3. Optimization

The Genetic Algorithm (GA) is used to systematically optimize the critical training hyperparameters of the proposed ConvNeXtV2-enhanced YOLOv8 model, aiming to maximize validation AP50 and prevent overfitting.

Inspired by biological evolution, the genetic algorithm is fundamentally an optimization algorithm that begins with a population of a certain size N [26,27]. The genetic algorithm is an effective algorithm for solving complex and multi-dimensional problems. In the problem-solving process, the fitness values of each individual in the population is evaluated using the fitness function. This function can be tied to either a single objective function or a multi-objective function [28]. The fitness function is generally defined as shown in Equation (9):

(9) $${FF}_{x}=\frac{f_{x}}{\bar{f}},$$

Here, the fitness value of individual *x* is ${FF}_{x}$, the objective function value $f_{x}$ calculated for *x* and *f* is an average of the objective function values calculated for all individuals in the population. As a second step, the next generation is created with the calculated fitness values, evolving from the solutions in the current generation (currentGen). This process enters a cycle until the specified number of generations (Genmax) or the specified stopping criteria are met. In the new generation creation phase, the selection operators integrated in the genetic algorithm ensure the selection of individuals with high fitness values. In this way, it helps the algorithm to reach the best solution. Roulette wheel selection, which is one of the selection operators, is one of the fitness proportional selection techniques. This method calculates the probability of selection for each individual and selects those with high probability into the parent pool [27,29]. The selection probability is calculated as in Equation (10).

(10) $$p_{i}=\frac{f_{i}}{\sum_{x=1}^{N}f_{x}},$$

Here, $f_{i}$ is the fitness value of the ith individual, N is the population size and $f_{x}$ is the sum of the fitness values of all individuals in the population. Basically, the genetic algorithm is defined as in Algorithm 1 [28].
**Algorithm 1** Typical genetic algorithm//Genetic algorithm parameters
//Pop (0) ← Generate initial
//Genmax ← Maksimum generation
//currentGen ← 1 initial value
**while** currentGen < Genmax **do**
   Evaluate Pop(currentGen-1)#*Pop(currentGen-1): the fitness value is calculated with the help of the fitness function.*   Produce Pop(currentGen) depends#*Pop(currentGen): implementation of selection operators for parents selection*     on Pop(currentGen-1)
   currentGen ← currentGen + 1
**end while**


During the genetic algorithm (GA) optimization process, the key hyperparameters affecting the training stability and detection performance of the proposed model were identified and evaluated across a wide search space. Table 1 presents the hyperparameters and search ranges used in the optimization.

The hyperparameters listed in Table 1 directly impact the model’s convergence behavior, learning rate, level of regularization, and stability at the start of training. Thus, the GA evaluated various combinations of the initial learning rate, momentum, weight decay, and warmup parameters to determine the most suitable set of hyperparameters. The optimal values obtained and their effects on model performance are presented in detail in the Section 3.

## 3. Results

The experiments were conducted in three main phases: evaluation of individual models, evaluation of ensemble models, and evaluation of the optimized ensemble approach. The performance results based on these phases are detailed in the subheadings.

### 3.1. Dataset

**Figure 2:**
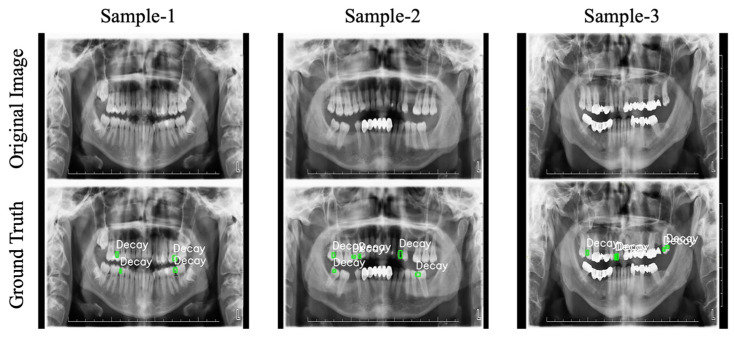
Sample Original Panoramic Radiographs from the Dataset and Corresponding Ground Truth Images to Expert Labels.

Approval for this study was obtained from the Clinical Research Ethics Committee of the Mersin University Rectorate (approval date: 30 October 2024; decision no.: 2024/1047). The dataset used in this study was compiled from panoramic dental radiographs obtained from the Faculty of Dentistry at Mersin University. The dataset contains 474 images with a resolution of 540 × 380 pixels. The images were selected to represent varying locations, sizes, and appearance characteristics of carious areas. Experts annotated the caries areas in the dataset using the bounding box method and converted the labels to YOLO format. Annotation and data preprocessing were performed using the Roboflow platform to ensure the images were transformed into a standardized, consistent format suitable for model training. During the labeling process, each carious lesion was delineated to ensure that the model would focus on the relevant pathological areas during training and evaluation. This approach allows for the localization of carious lesions without requiring pixel-level segmentation. Given the overlap of anatomical structures, low contrast, and limited visibility of small lesions in panoramic radiographs, bounding box-based labeling is a practical and feasible data preparation strategy for this study. The dataset was used to evaluate the performance of the proposed YOLOv8-based model in detecting and localizing caries. Labeled example images are presented in Figure 2. The figure shows the original panoramic radiographs alongside the ground truth bounding box annotations created by experts.

### 3.2. Performance Metrics

This study used the Box Precision (Box(P)), Recall (R), and AP50 metrics to evaluate the performance of the proposed object detection model. Together, these metrics assess the model’s ability to generate true positive predictions, its capacity to capture regions of interest, and its overall localization performance.

- Box precision indicates the proportion of positive predictions that are correct. It is calculated as the ratio of true positive detections that meet a specific intersection over union (*IoU*) threshold to the total number of positive predictions:

(11) $$Box(P)=\frac{TP}{TP+FP},$$

Here, (*TP*) refers to true positive detections, while (*FP*) refers to false positive detections. In terms of tooth caries detection, a high Box(P) value indicates that the areas the model flags as caries largely overlap with actual carried areas. This is important for reducing the risk of unnecessary clinical examinations or false alarms.

- Recall is a metric that indicates the proportion of actual caries areas that the model can detect. Mathematically, it is expressed as follows:

(12) $$R=\frac{TP}{TP+FN},$$

Here, *FN* indicates regions that experts labeled as carious but which the model did not detect. A high recall value means the model is less likely to miss carious regions. In clinical decision support systems, recall is a critical metric, especially for identifying small or low-contrast lesions that could be overlooked otherwise.

- AP50 is the average of the average precision (*AP*) values calculated for each class when the intersection over union (*IoU*) threshold is set to 0.5. This metric provides a single value that allows for the evaluation of the model’s performance in both classification and localization:

(13) $${mAP}_{50}=\frac{1}{C}\sum_{C=1}^{C}{AP}_{c}\;(IoU\geq 0.5),$$

Here, *C* represents the total number of classes, and ${AP}_{c}$ denotes the area under the precision–recall curve for the relevant class. Since the goal of this study is to detect carried regions, the AP50 value indicates the model’s overall detection performance at the specified intersection-over-union (*IoU*) threshold.

Generally, Box(P) was used to control for false positive predictions, recall to measure the rate of detection of actual carried regions, and AP50 to evaluate the model’s overall detection and localization performance. Together, these three metrics provide a more balanced analysis of the proposed model’s detection behavior in clinical images.

### 3.3. Quantitative Results and Comparative Peformance Analysis

In this study, the performance of three different models was comparatively evaluated for the detection of dental caries in panoramic radiographs: (i) the baseline YOLOv8 model, (ii) the proposed model enhanced with ConvNeXtV2 blocks but without hyperparameter optimization, and (iii) the proposed model optimized using a Genetic Algorithm (GA). To ensure a fair comparison, all models were analyzed using the same data split, the same test set, and the same evaluation metrics. During the testing phase, 47 panoramic images and a total of 153 caries regions present in these images were used. Performance evaluation was based on Box Precision (Box(P)), Recall (R), F1-score, and AP50 metrics. In addition, 95% confidence intervals were calculated using 1000 bootstrap resampling iterations to quantify the uncertainty of the reported performance values.

An examination of the results presented in Table 2 reveals that the baseline YOLOv8 model achieves relatively high precision with a Box(P) value of 0.777 (95% CI: 0.694–0.864), but its recall value of 0.477 (95% CI: 0.404–0.572) limits its ability to detect a significant portion of actual caries lesions. This imbalance between precision and recall is also reflected in the F1-score, which remains at 0.591 (95% CI: 0.517–0.675) for the baseline model, indicating that the trade-off between false positives and false negatives has not yet been optimally balanced. This result indicates that the baseline YOLOv8 model can control false-positive predictions to a certain extent but fails to achieve sufficient sensitivity, particularly in detecting low-contrast or small carious lesions. Overlapping anatomical structures, low resolution, and the uncertainty of lesion borders in panoramic radiographs can be considered among the primary causes of this limitation.

In the proposed model enhanced with ConvNeXtV2 blocks, the recall value increased from 0.477 to 0.549 (95% CI: 0.458–0.603), and the F1-score improved accordingly from 0.591 to 0.625 (95% CI: 0.548–0.689). This increase demonstrates that the ConvNeXtV2 blocks contribute to the model’s ability to detect defective regions, and that this contribution is substantial enough to yield a better overall balance between precision and recall, despite the individual decline in Box(P). However, the decrease in the Box(P) value from 0.777 to 0.725 (95% CI: 0.669–0.839) suggests that while the model generates more positive predictions, there has been an increase in the number of false positive predictions. It is also worth noting that the confidence intervals of the baseline and proposed models overlap considerably for most metrics, suggesting that some of these differences should be interpreted with caution given the limited sample size (47 images, 153 lesions). Therefore, it is observed that while architectural improvements provide an advantage in terms of recall and F1-score, they create a need for additional optimization regarding the precision–recall balance.

To statistically compare case-level prediction correctness across the same independent test set, pairwise comparisons were performed using two-sided exact McNemar tests. No statistically significant difference was observed between the baseline YOLOv8 and the proposed ConvNeXtV2-enhanced model, although the proposed model corrected more baseline errors than vice versa (15 versus 8 discordant cases; exact *p* = 0.210). Similarly, the comparison between the baseline YOLOv8 and the optimized proposed model was not statistically significant (12 versus 7 discordant cases; exact *p* = 0.359). No statistically significant difference was found between the proposed and optimized proposed models either (10 versus 12 discordant cases; exact *p* = 0.832). Thus, while the proposed models achieved numerically higher F1-score and AP50 values, the paired case-level analysis did not provide sufficient statistical evidence to conclude that their prediction correctness differed significantly on the current test set.

To improve this balance, the training hyperparameters of the proposed model were optimized using GA. As a result of the optimization, the initial learning rate (lr0) was set to 0.00705, momentum to 0.91218, and weight_decay to 0.0005. Additionally, warmup_epochs was set to 2.8182, warmup_momentum to 0.79129, and warmup_bias_lr to 0.1025. This combination of hyperparameters contributed to the model exhibiting more stable convergence during the training process and reducing the tendency for false positive predictions.

The proposed model, optimized using GA, achieved the highest precision and overall detection performance with Box(P), F1-score, and AP50 values of 0.784 (95% CI: 0.724–0.908), 0.637 (95% CI: 0.541–0.714), and 0.626 (95% CI: 0.498–0.687), respectively. The increase in the AP50 value from 0.539 (95% CI: 0.436–0.622) in the baseline YOLOv8 model to 0.626 in the optimized model demonstrates that the proposed approach improves localization and overall object detection performance, while the rise in F1-score from 0.591 to 0.637 further confirms that the optimized model achieves the most favorable balance between precision and recall among the three configurations. However, the optimized model’s recall value is 0.536 (95% CI: 0.426–0.607), which is only slightly lower than that of the unoptimized proposed model, and the overlapping confidence intervals between these two recall values indicate that this difference is not substantial. This indicates that GA optimization provides a more balanced detection performance by increasing precision, F1-score, and AP50 values rather than maximizing the recall value alone.

Compared to the standard YOLOv8 model, the optimized proposed model achieved higher values for all Box(P), recall, F1-score, and AP50 metrics. Notably, the confidence intervals for Box(P) and AP50 between the baseline and optimized models show minimal overlap, supporting the reliability of the observed improvements in these two metrics. However, when compared to the unoptimized proposed model, while there is a slight decrease in recall, significant improvements are observed in Box(P), F1-score, and AP50 values. This result demonstrates that the combined use of ConvNeXtV2-based architectural improvements and GA-based hyperparameter optimization has made the model’s overall detection behavior more balanced, as reflected consistently across the F1-score metric.

Overall, the ConvNeXtV2 blocks enhanced the model’s ability to detect caries regions, while the subsequent GA-based optimization further improved the balance between precision, recall, and localization performance. Among the three evaluated configurations, the optimized proposed model achieved the highest F1-score, AP50, and Box(P) point estimates, demonstrating the most balanced overall detection performance. The pairwise McNemar analysis showed that the proposed models produced favorable error-correction patterns relative to the baseline model, although the differences did not reach statistical significance on the current independent test set. Considering the limited number of test images, these findings should be interpreted together with the consistent improvements observed across the complementary performance metrics and bootstrap estimates. Overall, the results support the practical potential of the proposed framework and justify its further validation using larger, independent, and more diverse multi-center datasets.

### 3.4. Ablation Study on ConvNeXtV2 Block Placement

To justify which C2f modules received ConvNeXtV2 blocks, an ablation study was conducted by progressively integrating ConvNeXtV2 blocks into the C2f modules corresponding to different feature pyramid stages of the detection head (P3, P4, P5). Each variant was trained and evaluated under identical conditions (same data split, same test set) to isolate the effect of block placement on detection performance and model complexity. Table 3 summarizes the results.

Placing ConvNeXtV2 blocks only at the highest-resolution stage (P3) led to a complete failure of convergence (precision, recall, and AP50 all reduced to zero), most likely because the large receptive field and channel reweighting introduced by the ConvNeXtV2 block at this early, high-resolution stage disrupted the gradient flow before the network could learn stable low-level features. This configuration also nearly doubled the parameter count relative to the baseline (84.6 M vs. 43.6 M) and increased inference time from 6.40 to 13.11 ms/image (156.2 to 76.3 FPS), so the added computational cost did not translate into any detection benefit. Extending the integration to P3 + P4 restored stable training and improved AP50 over the baseline (0.575 vs. 0.539), at a further modest increase in latency (14.67 ms/image, 68.1 FPS), confirming that combining ConvNeXtV2 blocks across multiple scales is necessary for stable optimization. Adding the P5 stage (P3 + P4 + P5) further improved precision, recall, and AP50 (0.799/0.536/0.593), indicating that enriching semantic, low-resolution features benefits localization of low-contrast caries regions the most, at the cost of roughly 2.8× the baseline parameter count (122.6 M) and an inference time of 15.36 ms/image (65.1 FPS). Notably, this final latency increase over the P3-only variant was only 2.25 ms (13.11 to 15.36 ms) despite resolving the convergence failure entirely and raising AP50 from 0.000 to 0.593, indicating that the additional runtime cost of full multi-scale integration is modest relative to the accuracy and stability gained. Based on this trade-off between accuracy gain and computational cost, the P3 + P4 + P5 configuration was selected as the backbone for the proposed architecture. This configuration was subsequently combined with GA-based hyperparameter optimization to obtain the final model, whose performance is reported in Table 2.

### 3.5. Visual Results

**Figure 3:**
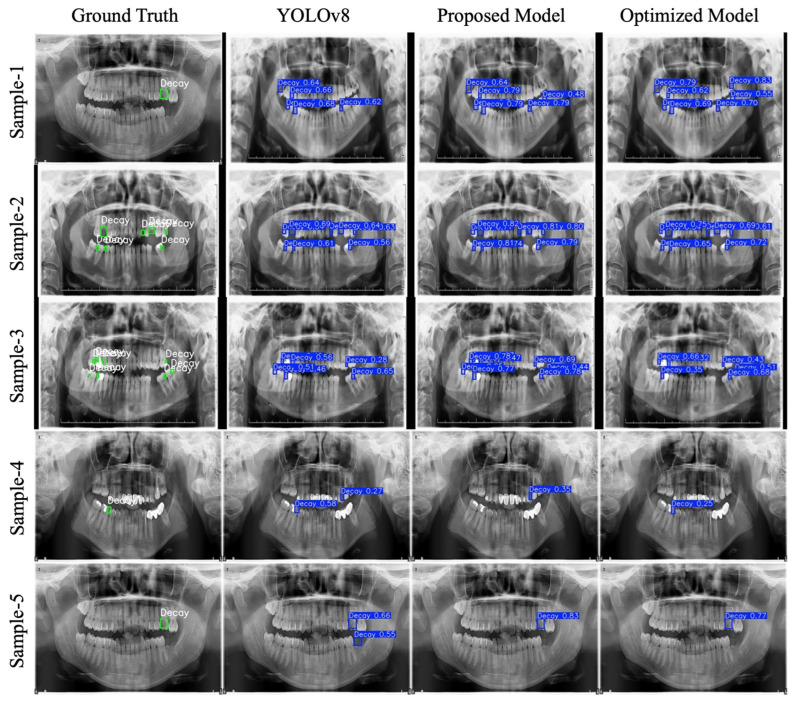
Comparative detection results for five representative panoramic radiographs. From left to right, the columns show expert ground-truth annotations (green bounding boxes), YOLOv8 predictions, predictions of the ConvNeXtV2-enhanced proposed model, and predictions of the GA-optimized model (blue bounding boxes with confidence scores).

To qualitatively evaluate model performance, detection results obtained from panoramic radiography samples representing different levels of clinical difficulty were analyzed comparatively. In this context, each model’s ability to identify carious areas, localization accuracy, and prediction reliability were evaluated using five different case examples. The comparison was conducted using ground truth annotations created by expert dentists, the standard YOLOv8 model, the proposed model enhanced with ConvNeXtV2 blocks, and the proposed model optimized using a Genetic Algorithm. The visual results are presented in Figure 3.

The ground truth annotations shown in Figure 3 indicate the reference locations of the carious regions identified by experts. Upon examining the results from the standard YOLOv8 model, it is observed that while the model can detect distinct carious areas, its performance is limited in regions containing low-contrast, small-scale, or anatomically overlapping structures. In some cases, the failure to detect lesions or the variability in confidence scores indicates that the standard YOLOv8 model may struggle to distinguish fine pathological details in panoramic radiographs.

The proposed model, enhanced with ConvNeXtV2 blocks, tends to detect more lesion areas in visual results compared to the standard YOLOv8. This suggests that ConvNeXtV2 blocks contribute to the extraction of more discriminative features in low-contrast and morphologically ambiguous regions. However, consistent with the quantitative results, an increase in false positive predictions is observed in the proposed model in certain cases. This finding supports the notion that while the architectural improvement increases the recall value, it creates a need for additional balancing in terms of precision.

The visual outputs of the proposed model optimized using GA generally exhibit more balanced detection behavior. Following optimization, it is observed that the model reduces some false positive predictions and that the detected carried regions align more consistently with the ground truth annotations. Particularly in cases involving complex anatomical structures, the optimized model’s ability to produce more selective predictions demonstrates that GA-based hyperparameter optimization contributes to the model’s prediction stability. However, it should be noted that some small or low-contrast lesions may still be missed.

When the comparison between models is evaluated as a whole, it is observed that switching from the standard YOLOv8 model to the proposed ConvNeXtV2-based model enhances the ability to detect rotten regions, while GA optimization balances this improvement with higher precision and AP50 values. These visual findings, consistent with the quantitative results, demonstrate that the optimized proposed model offers a more stable and balanced detection performance compared to the baseline YOLOv8.

In conclusion, qualitative analyses demonstrate that the proposed approach has the potential to serve as a supportive AI method for caries localization in panoramic radiographs. However, the visual results also indicate that the model still has room for improvement, particularly in small, low-contrast, or anatomically complex regions. Therefore, the proposed model should be considered, in its current form, not as an independent diagnostic system but as an auxiliary decision-support tool to assist dentists in their evaluation process.

## 4. Discussion

### 4.1. Comparison of Previous Studies with Proposed Model

In this study, we propose a YOLOv8-based object detection model enhanced with ConvNeXtV2 blocks and optimized using a Genetic Algorithm (GA) for the detection of dental caries in panoramic radiographic images. Experimental results show that the GA-optimized model achieved a Box Precision of 78.4%, a Recall of 53.6%, and an AP50 of 62.6%. These results demonstrate an improvement, particularly in terms of Box Precision and AP50, when compared to the standard YOLOv8 model. However, the fact that the recall value remains at a moderate level indicates that the model may still miss some carried areas and is open to further improvement in this regard.

In the literature, deep learning-based studies on dental caries detection can generally be categorized into three main groups: image- or dental impression-based classification approaches, pixel-level segmentation methods, and bounding box-based object detection models. Since these approaches utilize different data types, labeling schemes, and evaluation metrics, direct numerical comparison of results is limited. Therefore, in this section, previous studies are discussed with consideration of task type and clinical output format.

- In classification-based studies, the model typically aims to detect the presence of cavities in an image or a cropped region of a tooth. For example, İmak and colleagues reported an accuracy of 99.13% using a multi-input-layer CNN architecture on periapical images [10]. Khan and colleagues achieved 98.99% accuracy using the VGG16 model [6]. Oztekin and colleagues, on the other hand, reported 92% accuracy using the ResNet-50 model on dental patches derived from panoramic images and visualized the model’s decisions using Grad-CAM heatmaps [11]. Haghanifar and colleagues also achieved an accuracy of 86.05% using a classification approach based on dental segmenting and capsule networks [13]. While these studies offer high classification accuracy, they generally do not directly provide the exact location of the cavity at the bounding box level. Therefore, the use of classification models in clinical decision support may require additional explainability or localization methods regarding lesion localization.
- Segmentation-based approaches provide more detailed localization information by enabling the identification of pathological areas at the pixel level. Casalegno and colleagues achieved a 72.7% IoU using CNN-based semantic segmentation on near-infrared transillumination images [4]. Lian and colleagues, on the other hand, reported an IoU score of 0.785 for caries detection using an nnU-Net-based approach on panoramic images [5]. Although segmentation methods can provide detailed boundary information, they require high-quality pixel-level labels. The unclear boundaries of cavities in panoramic radiographs, overlapping anatomical structures, and inter-expert interpretation differences can complicate the process of creating pixel-level reference labels. From this perspective, bounding box-based object detection can be considered a practical alternative that offers clinically usable localization information with a lower labeling burden compared to segmentation.
- In object detection-based studies, the goal is to locate carried areas on the image. Zhang and colleagues used an SSD-based model to analyze intraoral photographs [7]. Thanh and colleagues, on the other hand, reported a sensitivity of 87.4% for carious lesions using YOLOv3 on intraoral images captured with smartphones [12]. However, intraoral photographs provide a different imaging context than panoramic radiographs because they contain visual cues such as color, texture, and surface irregularities. Panoramic radiographs, on the other hand, are grayscale images with low contrast and a high degree of anatomical superimposition. For this reason, detecting caries in panoramic radiographs can be considered a more challenging task, particularly when it comes to small and poorly defined lesions.

The recall value of 0.477 for the standard YOLOv8 model used in this study demonstrates that these challenges in panoramic radiographs directly affect model performance. The increase in the recall value to 0.549 following the integration of ConvNeXtV2 blocks indicates that this architectural improvement contributes to the model’s ability to detect carious areas. However, the decrease in the Box Precision value at this stage revealed that while the model generated more positive predictions, the tendency for false positives also increased. The subsequent rise in the Box Precision value to 0.784 and the AP50 value to 0.626 following GA-based hyperparameter optimization indicates that the optimization process improved the model’s overall detection balance.

Overall, the main contribution of the proposed approach is that it combines YOLOv8’s object detection capabilities with ConvNeXtV2-based feature extraction and GA-based hyperparameter optimization for caries localization in panoramic radiographs. The results obtained demonstrate that the proposed model offers more balanced performance compared to standard YOLOv8. However, since the recall value still has room for improvement, it is more appropriate to consider the model not as an independent diagnostic system for clinical applications, but as an auxiliary decision-support component that can assist dentists in their evaluation process.

### 4.2. Performance Effects of Confidence Threshold

**Figure 4:**
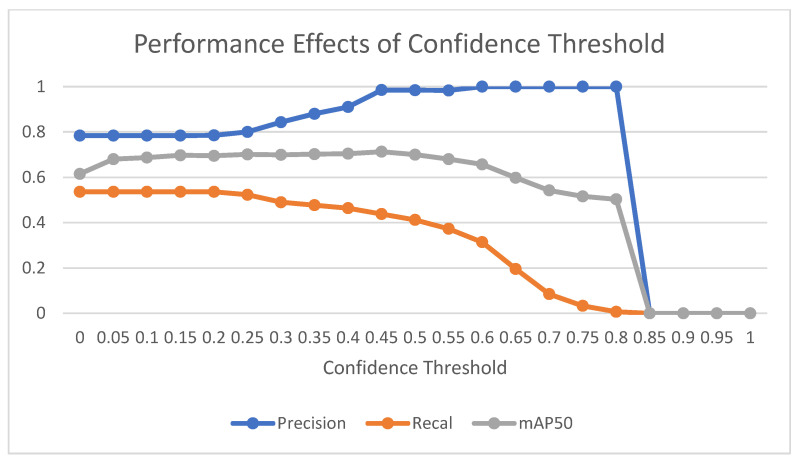
The effect of the confidence threshold on precision, recall, and AP50.

In object detection models, the confidence threshold is a key parameter that determines whether predicted bounding boxes are accepted or rejected. When this value is set low, the model generates more predictions; however, there is a risk of an increase in false positives. Conversely, at high threshold values, only more reliable predictions are retained; however, some genuine outliers may be missed. Therefore, the behavior of the proposed model under different confidence threshold values was analyzed using precision, recall, and AP50 metrics. The results are presented in Figure 4.

Examining Figure 4, it can be seen that the model generates more predictions at lower threshold values, and consequently, the recall value remains relatively high. Specifically, in the 0.00–0.20 range, recall hovers around 0.53. This indicates that the model maintains its ability to detect defective regions. However, the fact that precision remains at approximately 0.78–0.79 within the same range suggests that some predictions with low confidence scores are being included in the model as false positives.

As the threshold value rises above 0.25, precision increases while recall gradually decreases. This result indicates that the model has become more selective and is filtering out low-confidence predictions. Within the 0.40–0.55 range, precision reaches quite high values, but recall drops significantly. This situation highlights the precision–recall trade-off commonly observed in object detection models.

It is observed that the most balanced performance in terms of the AP50 metric is achieved within the threshold range of approximately 0.30–0.50. Within this range, the model is able to both reduce false positives and detect cavities with sufficient accuracy. In contrast, at threshold values of 0.60 and above, recall decreases rapidly, leading to a decline in overall detection performance. At values of 0.85 and above, the model produces almost no valid predictions, and the metrics approach zero.

Overall, it can be said that the most appropriate confidence threshold range for the proposed model is 0.30–0.50. In clinical scenarios where more reliable predictions are prioritized, the 0.45–0.50 range may be preferred, whereas in situations where avoiding the omission of carious areas is more critical, the 0.30–0.35 range may be more appropriate. Therefore, the confidence threshold value must be carefully determined based on the intended use.

### 4.3. Limitations

**Figure 5:**
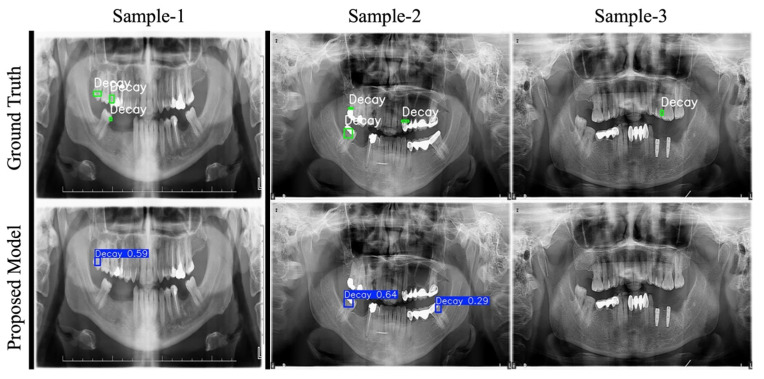
Sample ground truth and prediction results illustrating the limitations of the proposed model. Green bounding boxes indicate ground-truth annotations, whereas blue bounding boxes indicate model predictions with the corresponding confidence scores.

Although the proposed approach offers more balanced detection performance compared to standard YOLOv8, it has certain limitations stemming from the nature of panoramic radiographs and the scope of the dataset. Low contrast, anatomical overlap, and limited visibility of small lesions can lead to missing or incorrect predictions in some cases. Examples illustrating these limitations are presented in Figure 5.

Figure 5 compares the ground truth annotations with the predictions of the proposed model. In Sample-1, although there are multiple carried areas on the ground truth, only one region was detected. In Sample-2, in addition to a correct detection, an additional prediction with a low confidence score that does not fully align with the ground truth was generated. In Sample-3, a small and low-contrast carried area was missed. These examples demonstrate that the method has room for improvement, particularly in small, low-contrast, and anatomically complex areas.

The main limitations of this study are summarized below:

- Small and early-stage cavities: As seen in Sample-3, small lesions with low contrast may not be detectable. This limitation may be related to the resolution of panoramic radiographs and the limited representation of small lesions in the dataset.
- Images containing multiple lesions: Sample-1 demonstrates that adjacent or similar-looking carious areas may not be fully captured in the same image.
- False-positive predictions: The additional prediction observed in Sample-2 suggests that structures around the root, restoration areas, or changes in radiographic density may mimic caries-like features.
- Low contrast and superimposition: The fact that carious tissue and surrounding anatomical structures exhibit similar gray-scale characteristics makes it difficult to distinguish their borders, particularly in areas where the root, adjacent teeth, and bone structures overlap.

To quantify the extent of the small-lesion limitation, the test set (153 lesions) was stratified into three equally sized groups (51 lesions each) based on bounding-box area.

As shown in Table 4, all three models detect large lesions reliably (78.4–80.4% recall), and medium lesions moderately well (60.8–68.6%), but recall for small lesions remains critically low across all configurations (5.9–11.8%). While the ConvNeXtV2-enhanced architecture improves small-lesion recall over the baseline YOLOv8 (11.8% vs. 5.9%), GA-based hyperparameter optimization, despite improving overall precision, F1-score, and AP50, slightly reduces small-lesion recall relative to the unoptimized proposed model (7.8% vs. 11.8%). This indicates that the optimization process, which favors overall precision–recall balance and reduces false positives, may implicitly deprioritize the detection of small, low-contrast lesions that are inherently harder to localize with confidence. This finding reinforces that the model’s overall recall of 53.6% is not uniform across lesion sizes, and that its clinical utility is currently concentrated on medium and large lesions rather than the small, early-stage lesions where early detection matters most.

Given these limitations, the proposed approach should be considered, in its current form, not as a standalone diagnostic system but as an auxiliary decision-support tool to assist dentists in their evaluation process. Future studies should evaluate broader and more diverse datasets, a more balanced representation of small lesions, external validation using panoramic radiographs obtained from different devices, and methods to improve resolution. Furthermore, while our framework utilizes bounding boxes to provide direct spatial interpretability, future studies could explore attention-based visualization techniques or post-hoc interpretability methods to further enhance the transparency of the model’s focus on subtle pathological features, thereby potentially increasing clinician trust in AI-assisted diagnosis.

### 4.4. Comparison with Other State-of-the-Art Detectors

To further contextualize the performance of the proposed model, we additionally trained and evaluated four recent object detectors, YOLOv9-C, YOLOv10-L, YOLO11-L, and Faster R-CNN, using the same data split and test set as the models reported in Table 2.

As shown in Table 5, the optimized proposed model achieved the highest precision (0.784) and AP50 (0.626) among the five compared detectors. Faster R-CNN obtained the highest recall (0.614), but its precision (0.369) was substantially lower than that of all other detectors, indicating a stronger tendency toward false-positive predictions; consequently, its overall AP50 (0.513) remained the lowest among the compared models. YOLO11-L achieved a slightly higher recall than the optimized proposed model (0.558 vs. 0.536) but a lower AP50 (0.566 vs. 0.626), suggesting a less favorable precision–recall balance. YOLOv9-C and YOLOv10-L, which are considerably more parameter-efficient (25.3 M and 25.7 M parameters, respectively) than the ConvNeXtV2-enhanced proposed architecture (122.6 M parameters), achieved AP50 values of 0.602 and 0.550, both lower than the optimized proposed model. Overall, these results suggest that the proposed model offers a more favorable balance between false-positive control and localization performance than the compared detectors on this dataset, at the cost of a considerably larger parameter count. Given the limited size of the test set (47 images, 153 lesions), this comparison should nonetheless be interpreted with the same statistical caution applied elsewhere in this study.

## 5. Conclusions

In this study, a YOLOv8-based object detection model, enhanced with ConvNeXtV2 blocks and optimized via Genetic Algorithm (GA), is proposed for the automatic detection of dental caries in panoramic radiographic images. Given the challenges of panoramic radiography such as low contrast, anatomical superimposition, and limited visibility of small lesions, this approach provides a practical framework for caries localization.

Experimental results demonstrate that the GA-optimized model achieved a Box Precision of 78.4%, a Recall of 53.6%, and an AP50 of 62.6%. These metrics represent an improvement over the baseline YOLOv8, particularly in reducing false positives and stabilizing the precision–recall balance. Size-stratified analysis further shows that this limitation is concentrated in small, early-stage lesions, where recall falls to 7.8–11.8% depending on configuration, compared to over 78% for large lesions. Given this, a recall of 53.6%, and in particular, the markedly lower recall observed for small lesions indicate that the proposed model is not yet ready for standalone clinical use and should currently be positioned strictly as a second-reader or decision-support tool used alongside, rather than in place of, expert dental examination. Consequently, the proposed approach should be evaluated as an AI tool supporting dentists’ decision-making processes rather than a standalone system.

Future work will focus on expanding the dataset to include diverse patient demographics and imaging devices, validating the model on external datasets, optimizing confidence thresholds for clinical utility, and evaluating real-time integration into clinical workflows. Additionally, adapting this architecture to periodontal disease detection, jaw lesions, and implant planning may further enhance the study’s clinical and technological contributions.

## Figures and Tables

**Table 1:** Hyperparameter Search Space Used in the Genetic Algorithm Optimization Process.

Hyperparameter	Search Range
lr0	(1 × 10^−5^)–(1 × 10^−1^)
momentum	0.60–0.98
weight_decay	0.00–0.01
warmup_epochs	0.0–5.0
warmup_momentum	0.0–0.95

**Table 2:** Comparative performance results of the models.

Model	Number of Images	Number of Lesions	Box(P) (95% CI)	R (95% CI)	F1-Score (95% CI)	AP50 (95% CI)
YOLOv8	47	153	0.777 (0.694–0.864)	0.477 (0.404–0.572)	0.591 (0.517–0.675)	0.539 (0.436–0.622)
Proposed Model	47	153	0.725 (0.669–0.839)	0.549 (0.458–0.603)	0.625 (0.548–0.689)	0.564 (0.491–0.638)
Optimized Proposed Model	47	153	0.784 (0.724–0.908)	0.536 (0.426–0.607)	0.637 (0.541–0.714)	0.626 (0.498–0.687)

**Table 3:** Ablation results for ConvNeXtV2 block placement.

Variant	Precision	Recall	AP50	Parameters	Inference (ms/Image)	FPS
Baseline	0.777	0.477	0.539	43.6 M	6.40	156.2
ConvNeXtV2 at P3 only	0.000	0.000	0.000	84.6 M	13.11	76.3
ConvNeXtV2 at P3 + P4	0.742	0.516	0.575	97.6 M	14.67	68.1
ConvNeXtV2 at P3 + P4 + P5	0.799	0.536	0.593	122.6 M	15.36	65.1

**Table 4:** Lesion size-stratified recall of YOLOv8, the proposed model, and the optimized proposed model.

Lesion Size Group	Area Threshold (px^2^)	YOLOv8 Recall	Proposed Model Recall	Optimized Proposed Model Recall
Small	≤73.5	5.9%	11.8%	7.8%
Medium	≤138.0	60.8%	68.6%	66.7%
Large	>138.0	78.4%	78.4%	80.4%

**Table 5:** Comparison with recent state-of-the-art object detectors on the same test set.

Model	Precision	Recall	AP50	Parameters
YOLOv9-C	0.780	0.510	0.602	25.3 M
YOLOv10-L	0.732	0.497	0.550	25.7 M
YOLO11-L	0.665	0.558	0.566	25.3 M
Faster R-CNN	0.369	0.614	0.513	41.3 M
Optimized Proposed Model	0.784	0.536	0.626	122.6 M

## Data Availability

The dataset used in this study is available on request from the corresponding author. The data is not publicly available due to privacy concerns.
